# Acute and Chronic Q Fever in a Child With Repaired Tetralogy of Fallot: A Case Report

**DOI:** 10.7759/cureus.88759

**Published:** 2025-07-25

**Authors:** Alaa Mohammed Al Juaid, Faisal Almalki, Reema E. Aloteibi, Thuraya Shaher Alharthi

**Affiliations:** 1 Pediatrics, King Abdullah Specialized Children Hospital, Ministry of National Guard - Health Affairs, Jeddah, SAU; 2 College of Medicine, King Saud Bin Abdulaziz University for Health Sciences, Jeddah, SAU; 3 College of Medicine, Taif University, Taif, SAU

**Keywords:** acute q fever, case report, chronic q fever, culture-negative endocarditis, pediatric q fever

## Abstract

In children, Q fever often presents as an acute febrile illness, sometimes with pneumonia or hepatitis. We report in this case a 38-month-old girl with complex congenital heart disease (tetralogy of Fallot, pulmonary atresia, and ventricular septal defect) who presented with a four-day history of fever, shortness of breath, and vomiting. She had prior cardiac interventions. After admission to the PICU, she was initially treated for severe pneumonia. During her stay, her condition worsened with heart failure, hepatosplenomegaly, and pancytopenia. After extensive investigations, Q fever serology was positive, indicating chronic Q fever. The patient was treated with doxycycline and hydroxychloroquine for 18 months. Her liver function improved, and echocardiography was normal after four months. This case highlights the importance of considering Q fever in pediatric patients with cardiac history, the need for comprehensive investigations, and appropriate long-term antibiotic treatment.

## Introduction

An outbreak of a febrile illness of unknown origin among abattoir workers in Australia led to the coining of the nomenclature “Q fever” by Derrick in 1937, where “Q” stands for “Query,” referring to the ambiguity around its cause [1]. Following investigations identified *Coxiella burnetii *(*C. burnetii*), a gram-negative intracellular bacterium, as the causative agent [2]. It is a zoonotic disease with a worldwide distribution, transmitted mainly through inhalation of aerosols from infected animals, such as goats and sheep, or through contact with contaminated animal products [3]. Q fever manifests in acute and chronic forms in both adults and children with distinct clinical presentations. In pediatrics, acute Q fever usually presents as a self-limiting febrile illness with symptoms such as headache, fatigue, and myalgia. Respiratory issues, including pneumonia, may occur, leading to symptoms like cough, chest pain, and respiratory distress [4]. On the other hand, severe acute manifestations are quite uncommon, such as hepatitis or myocarditis. These particularly can occur in immunocompromised or predisposed individuals [5]. Although rare in children, chronic Q fever is a severe condition often associated with endocarditis, especially in those with underlying cardiac conditions. This carries significant morbidity and mortality if untreated [6]. The highest risk category in pediatrics should be taken into consideration while making a differential diagnosis of culture-negative endocarditis, especially in patients with underlying cardiac illness. Furthermore, the transition from acute Q fever to chronic Q fever is extremely rare worldwide [4]. To our knowledge, very few cases of acute Q fever have been reported in Saudi Arabia.

In accordance with the CARE Guidelines [6], we report a case of a pediatric patient with a background of a complex congenital heart disease who presented with signs and symptoms of acute Q fever that transitioned to the chronic form within days of the initial illness.

## Case presentation

**Figure 1:**
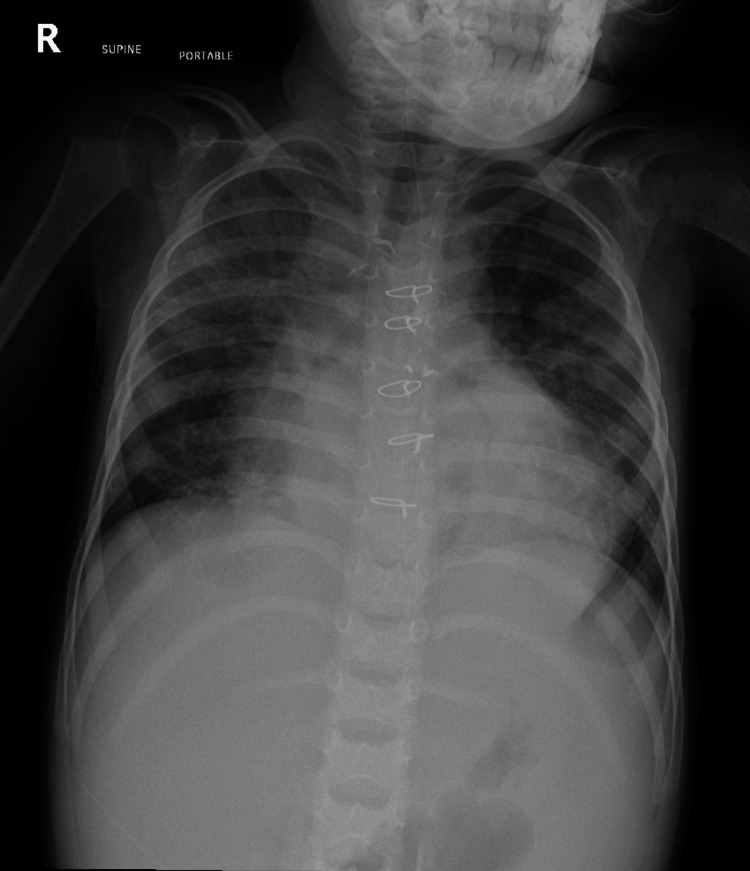
Chest X-ray taken at first presentation showing cardiomegaly, pneumonic changes, and increased pulmonary vascular margins.

Our patient, a 38-month-old full-term girl, is a known case of tetralogy of Fallot (TOF), pulmonary atresia, and ventricular septal defect. She was treated initially with a patent ductus arteriosus (PDA) stent at the age of two weeks. She then underwent Rastelli procedure at 16 months of age, which included an autologous right ventricle-to-pulmonary artery hand-sewn valved polytetrafluoroethylene (PTFE) conduit, size 16 mm. Prior to her presentation, she was clinically stable and only on aspirin. The patient presented to the emergency department of another hospital with a four-day history of fever, shortness of breath, and vomiting. The patient initially looked ill and irritable; she was in respiratory distress in the form of suprasternal and subcostal retractions, tachypneic (>40 breaths/min), and desaturating (<80% O_2_), with a temperature measured at 38 °C. The patient was admitted to the pediatric intensive care unit (PICU) and was started on non-invasive positive pressure ventilation and intravenous ceftriaxone, with an initial diagnosis of severe community-acquired pneumonia. Her initial chest X-ray showed evidence of cardiomegaly, increased pulmonary vascular margins, and pneumonic changes (Figure 1). The patient was then referred to our tertiary center and admitted to our PICU for further management and investigations.

Further investigation during the PICU stay revealed worsening of her baseline cardiac functions, in which she developed signs and symptoms of heart failure, and antibiotics were changed to meropenem and vancomycin. Transthoracic echocardiography (TTE) at that time showed a peak gradient of 70 mmHg of tricuspid regurgitation with pulmonary hypertension, for which she was managed by furosemide and sildenafil. Although no vegetations were found during the TTE, the presence of vegetation couldn’t be ruled out due to the presence of the PTFE conduit. Moreover, a few days into the PICU stay, she started to exhibit significant hepatosplenomegaly and bicytopenia, hemoglobin of 4.4 gram/dL, and platelet count of 73x109/L, which were not present during the initial illness. 

During the PICU stay, the patient's cardiac functions improved after three days on furosemide and sildenafil, with repeated echocardiography similar to her baseline, but the bicytopenia progressed to pancytopenia, with persistent hepatosplenomegaly. She received three packed red blood cells (RBCs) transfusions and one platelet transfusion during her stay. Two CT scans of the chest were done during the stay, but no evidence of vegetation was found. Both CT scans were correlated with the initial chest X-ray and the nonspecific evidence of community-acquired pneumonia; however, the patient still had a spiking fever despite being on antibiotics. All blood cultures for bacteria and fungi were negative.

On day 15 of the illness, her respiratory status improved; she was weaned off to a high-flow nasal cannula. On day 20, she was moved to the general ward after weaning to room air; at this time, she had completed a full course of meropenem and vancomycin and was not febrile anymore, but still had persistent hepatosplenomegaly. Multiple services were involved, and different laboratory and serological testing were conducted, but all were unremarkable.

On day 21 of her illness, her pancytopenia improved, but a new trend of transaminitis was noted. Her initial alanine aminotransferase (ALT) and aspartate aminotransferase (AST) were 93 unit/L and 91 unit/L, respectively. Both ALT and AST kept increasing during the admission until reaching 608 unit/L and 422 unit/L, respectively.

At that time, the serology for Q fever was sent and returned positive, with markedly elevated titers: phase II *C. burnetii* IgG >16,284 (normal <800), phase II IgM positive, phase I IgG >16,264, and phase I IgM also positive, findings indicating chronic Q fever. Further history-taking at the time of suspicion of Q fever revealed that the patient lives in a rural area with an extensive exposure to a farm of cattle and sheep less than five meters away. 

The patient was started on a combination of doxycycline and hydroxychloroquine, for a total of at least 18 months with regular outpatient clinic follow-up. After three days of starting the treatment, the ALT and AST started to trend down, until normalized after three months of treatment. The patient was discharged home in good health to complete her antibiotics course with serial outpatient clinic follow-ups. During the four months of treatment and follow-ups, her hepatosplenomegaly was improving gradually until it resolved, except for mild hepatomegaly of 1 cm below the costal margin. The repeated echocardiography after four months of therapy was unremarkable and consistent with her baseline echocardiography before the illness. The therapy side effects and duration were discussed with the parents, and the patient was compliant for up to four months of therapy, with no notable side effects. The patient is set to complete her therapy by at least 18 months, with monitoring of the IgG phase II *C. burnetii *antibody titers. Upon further discussion with the cardiology team, they preferred that the conduit be removed and the patient referred back for possible conduit removal by cardiac surgery mid-therapy or if symptoms developed. 

## Discussion

Our patient presented with a combination of all presentations of acute Q fever in pediatrics, such as the flu-like illness preceding her presentation to the emergency department. However, it is uncertain if it was associated with the onset of Q fever or just a form of common cold. Her presentation with pneumonia, on the other hand, was indeed suspicious for Q fever, as it did not improve on antibiotic treatment. Although the elevation of liver enzymes is not specific nor sensitive to this condition, it is considered a characteristic of the disease when combined with the patient’s risk factors and the course of pneumonia [3].

Even though rarely reported, chronic forms of the disease in children develop through culture-negative endocarditis, chronic hepatitis, or multifocal osteomyelitis [3]. Moreover, those with cardiac predisposition, such as those with valvulopathies, are at a higher risk for developing chronic Q fever. Although patients typically transition from the stage of acute Q fever to the chronic Q fever form within a short period, our patient transitioned from the acute Q fever form immediately to the chronic Q fever form. However, this transition was only biochemically evident in the phase I *C. burnetii* IgG, which was positive. Quick deterioration can happen in chronic stages that are delayed in diagnosis and can often lead to fatality. Cases reported before 1987 had a 37% mortality rate. However, recently, a mortality rate of 10% was seen in cases diagnosed in a matter of six months post-initial infection [7]. In terms of immunology, Q fever has acute and chronic stages that are correlated to two antigenic phases of antibody response. In an acute infective state, an antibody response, phase II antigen, is higher in response to a *C. burnetii* infection. On the other hand, an elevated phase I immunoglobulin G (IgG) titer corresponds to a chronic infection [3]. Additionally, symptoms of acute Q fever occur within two to three weeks of exposure to dust or aerosols that are contaminated with excreta from infected animals [3, 7].

Many experts have suggested that Q fever infection follows the pattern of *Mycobacterium tuberculosis* (TB), wherein after the acute infection, a patient may be seropositive and labelled as latent TB. Similar terminology has been suggested for Q fever, in which patients are labelled as persistent Q fever instead of chronic Q fever. This classification allows for the accurate identification of patients who exhibit biochemical evidence of Q fever but do not progress to its chronic form, which most commonly presents as culture-negative endocarditis in pediatric patients with valvopathy. Moreover, Q fever endocarditis is considered an indolent form of infective endocarditis; thus, the infected child could be mildly symptomatic for several years before a diagnosis is established, or could have a persistent Q fever that may not necessarily progress to chronic endocarditis if appropriately treated. Evidence of a well-defined latent phase of Q fever is not recognized yet, although a latent period of up to 20 years has been reported [7]. It is especially important to note that chronic infection develops exclusively in children with previous valvular disease and those who are immunocompromised. In fact, patients with valvulopathy and acute Q fever had a 38.7% chance of developing endocarditis, along with patients with valvular prostheses who posed the greatest risk of acquiring endocarditis [7].

According to the US Centers for Disease Control (CDC), doxycycline is the medication of choice for acute Q fever in children, in addition to another antibiotic. In children who are immunocompromised or have preexisting valvulopathies, doxycycline is also given for more than two weeks until resolution of symptoms [3].

At the time of suspicion for Q fever, serologic testing was performed to detect antibodies against *C. burnetii*, revealing a significantly elevated IgG phase II antibody titer >16,284 (normal <800) and positive IgM phase II antibodies, consistent with a robust immune response typically seen in acute Q fever or early chronic infection. Additionally, the IgG phase I antibody titer was >16,264 (normal <800), and IgM phase I antibodies were positive, strongly suggesting chronic Q fever, as phase I IgG titers ≥1:800 are a hallmark of chronic infection, particularly endocarditis.

These findings indicate a possible rapid progression from acute to chronic Q fever, an exceptionally rare occurrence within a single hospital admission. Further history-taking revealed that the patient, residing in a rural area of Saudi Arabia, had extensive exposure to a farm with cattle and sheep within a five-meter proximity, a significant risk factor for *C. burnetii* infection due to aerosol transmission from infected animals. A fourfold decrease in phase I IgG levels and the complete remission of phase II IgM titers is a recognized sign for discontinuing antibiotics after the exhibition of clinical improvement [7]. A serological follow-up every four months up to two years is also a recognized treatment approach, and can be extended up to five years because of the risk of relapse [7].

In the Netherlands, a study about the cost-effectiveness of a screening program for chronic Q fever varied greatly amongst different risk groups, and the results were dependent on the prevalence of chronic Q fever. Therefore, screening those with cardiac disease in areas with a high prevalence of chronic Q fever may be cost-effective [8]. As for vaccination of cattle, reduced shedding of *C. burnetii* in milk, placenta, and uterine fluid is well-established [9]. Moreover, effective Q fever control at the herd level requires 80% of animals to be vaccinated during the initial stage of infection [10]. There is a lack of evidence on vaccinating children living near cattle who are at a higher risk of acquiring the infection. Furthermore, the vaccine given today in Australia is targeted only toward people older than 15 years of age as per the CDC recommendations [11]. Saudi Arabia could perhaps be an endemic area for *C. burnetii *infections. According to a recent study done in Makkah, camels had the highest seroprevalence of 50.4%, while goats had 44.6% and sheep only had 36.8% [12]. However, whether camels are a source of infection remains uncertain. 

## Conclusions

This case emphasizes the significance of considering Q fever in pediatric patients, especially those with underlying heart conditions. The complex and often overlooked nature of Q fever manifestations was evident. Timely diagnosis through comprehensive serological testing was crucial. The successful treatment with doxycycline and hydroxychloroquine for an extended period, along with close monitoring, led to the patient's improvement. It also highlights the need for further research and awareness to better manage and prevent Q fever in similar high-risk populations.

## References

[1] Derrick EH (1983). "Q" fever, a new fever entity: clinical features, diagnosis and laboratory investigation. Rev Infect Dis.

[2] Maurin M, Raoult D (1999). Q fever. Clin Microbiol Rev.

[3] Anderson A, Bijlmer H, Fournier PE (2013). Diagnosis and management of Q fever--United States, 2013: recommendations from CDC and the Q Fever Working Group. MMWR Recomm Rep.

[4] Maltezou HC, Raoult D (2002). Q fever in children. Lancet Infect Dis.

[5] Tissot-Dupont H, Raoult D (2008). Q fever. Infect Dis Clin North Am.

[6] (2025). CARE statement. CARE guidelines. https://www.care-statement.org.

[7] Alhadhoud SA, Vel MT, Al Qbandi M (2018). Q fever endocarditis after right ventricle to pulmonary artery conduit insertion: Case series and review of the literature. Ann Pediatr Cardiol.

[8] de Boer PT, de Lange MM, Wielders CC (2020). Cost-effectiveness of screening program for chronic Q fever, the Netherlands. Emerg Infect Dis.

[9] Courcoul A, Hogerwerf L, Klinkenberg D, Nielen M, Vergu E, Beaudeau F (2011). Modelling effectiveness of herd level vaccination against Q fever in dairy cattle. Vet Res.

[10] Gisbert P, Hurtado A, Guatteo R (2024). Efficacy and Safety of an Inactivated Phase I Coxiella burnetii Vaccine to Control Q Fever in Ruminants: A Systematic Review. Animals (Basel).

[11] (2025). Victorian Department of Health. Q fever vaccination. CDC Yellow Book.

[12] Alkenani NA, Baroom HM, Almohimeed AA (2024). Serological investigation of Coxiella burnetii infection (Query fever) in livestock in Makkah Province, Saudi Arabia. Vet World.

